# Sanatorium Treatment for the Poor

**Published:** 1911-01-21

**Authors:** 


### SANATORIUM TREATMENT FOR THE POOR.

### WHAT THE CHARITY ORGANISATION SOCIETY IS DOING.

THERE is only one Society which is supplying sanatorium treatment for the poor in every part of London, the Charity Organisation Society, which, working through its forty-one district committees, sends every year an increasing number of patients to sanatoria. Last year over a hundred people were sent, this year the numbers are larger. The total number sent from 1902, when the work started, to December 31, 1909, was 385.

The method of working is, briefly, as follows: The applicant requires no recommendation beyond a good chance of recovery, a matter in which character and environment, as well as the condition of his lungs, have to be taken into account; he is examined and passed by a specialist in chest diseases, he goes with the minimum of delay to a sanatorium, where he is kept, as a rule, as long as the doctor wishes him to remain; his family is provided for while he is away, and on his return efforts are made to find him suitable work if his own occupation is unsuitable, or to improve the conditions under which he must work.

The following sanatoria are used by the Society: Kelling Sanatoria, Norfolk; Brompton Hospital Sanatorium, Frimley; Maitland Sanatorium, Peppard, Oxon.; Maltings Farm Sanatorium, Essex; and Boxgrove Cottage Sanatorium, Tilehurst. Six permanent beds are kept at Kelling Sanatorium. The average length of stay at the sanatorium is seventeen weeks and four days.

All the 385 patients sent away before December 31, 1909, were looked up early this year; from their own statements their condition was as follows: 50.12 per cent. were working, fit for work, or improved, and 11.42 per cent. were ill; we were informed that 26.49 per cent. were dead, and 11.94 per cent. could not be traced. It should be added that these results are not presented in an unduly favourable light: the figures are a bald statement of fact. It should be added also that the present medical referee did not begin his work till 1904, that the figures include cases sent to three sanatoria which no longer exist, that new methods bring constantly improving results, and that the results in the future are likely to be even better than in the past.

The results obtained justify, it is felt, the continuance of the work by the Society. But the financial position is a very serious one. The cost of the sanatorium work is shared by district committees and the Central Council of the Society. The district committee finds 15s. a week towards the cost of the patient's treatment, and has besides to find whatever money may be necessary to keep the family going. The money is raised on each case separately from relations who can help—if any—clergy, employers, local charities, etc. The medical sub-committee finds the balance of the cost of treatment—*i.e.* 10s. to 15s. a week. For this purpose over £900 a year has to be found; of this sum about £50 is received in regular subscriptions, and the remainder must be begged for.

The subject of tuberculosis is very much in the air nowadays, and much money is rightly being spent in spreading the knowledge of the prevention and treatment of the disease. But the Society feels, and others will feel, that however urgent propaganda may be, the actual curative and educational work should not be allowed to fail. The work of the C.O.S. is now well known to many hospitals and private practitioners; many institutions are regularly sending their early cases to the Society for sanatorium treatment, and the question now is, how can the Society be enabled to continue dealing with those cases?

